# Une forme particulière de pancytopénie

**DOI:** 10.11604/pamj.2018.29.209.14055

**Published:** 2018-04-09

**Authors:** Hafsa Chahdi, Mohamed Oukabli

**Affiliations:** 1 Service d’Anatomie Pathologique, Hôpital Militaire, d’Instruction Mohamed V, Rabat, Maroc

**Keywords:** Oxalose médullaire, pancytopénie, fibrose, Medullary oxalosis, pancytopenia, fibrosis

## Abstract

Primary hyperoxaluria is a rare disease whose incidence is estimated at less than 1 cases/million inhabitants/year. This is a congenital abnormality of hepatic metabolism leading to an endogenous overproduction of oxalate with excess urinary excretion. We report the case of a 43-year-old patient, was followed to end-stage renal disease hemodialysis, consulting for anemic syndrome with mucocutaneous pallor. Laboratory tests found pancytopenia with aplastic anemia. Bone marrow was difficult to achieve, bringing medullary blood hyperdilué and uninterpretable . Radiographs showed a homogeneous splenomegaly and small dedifferentiated a kidney marrow biopsy was performed. Histological examination revealed a myelofibrosis and birefringent crystals in polarized light, diagnosis retained: spinal oxalosis.

## Image en médecine

L’hyperoxalurie primitive est une pathologie rare dont l’incidence est estimée à moins de1 cas / million d’habitants/ an. Il s’agit d’une anomalie congénitale du métabolisme hépatique entrainant une surproduction endogène d’oxalate avec excès de son élimination urinaire. Nous rapportons le cas d’une patiente de 43 ans, qui était suivie pour insuffisance rénale terminale au stade d’hémodialyse, ayant consulté pour un syndrome anémique fait de pâleur cutanéo-muqueuse. Le bilan biologique a retrouvé une pancytopénie avec une anémie arégénérative. Le myélogramme était difficile à réaliser, ramenant un sang médullaire hyperdilué et ininterprétable. Le bilan radiologique a montré une splénomégalie homogène et des petits reins dédifférenciés. Une biopsie ostéomédullaire était pratiquée. L’examen histologique a mis en évidence une fibrose médullaire et des cristaux biréfringents en lumière polarisée, le diagnostic retenu est celui de l’oxalose médullaire.

**Figure 1:**
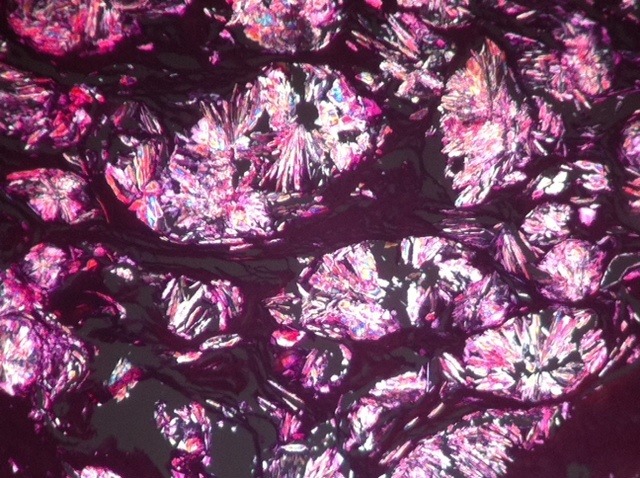
Aspect montrant la biréfringence des dépôts en lumière polarisée

